# Pharmacokinetics evaluation of newly formulated beads alginate/gum acacia loaded ketoconazole in rabbit plasma by oral administration

**DOI:** 10.5599/admet.2042

**Published:** 2023-11-30

**Authors:** Viviane Annisa, Teuku Nanda Saifullah Sulaiman, Akhmad Kharis Nugroho, Agung Endro Nugroho

**Affiliations:** 1 Program Doctoral Faculty of Pharmacy, Universitas Gadjah Mada, Indonesia; 2 Departement of Pharmaceutics, Faculty of Pharmacy, Universitas Gadjah Mada, Indonesia; 3 Departement of Pharmacology and Clinical Pharmacy, Faculty of Pharmacy, Universitas Gadjah Mada, Indonesia

**Keywords:** in vivo, drug, bioavailability, hydrogel, polymer

## Abstract

**Background and purporse:**

The combination of alginate and gum acacia in previous studies showed good results in inhibiting ketoconazole precipitation due to the supersaturation phenomenon. Ketoconazole-loaded alginate and gum acacia can produce hydrogel beads through cross-linking with Ca^2+^ using ionotropic gelation techniques. However, the pharmacokinetic study of the ketoconazole beads loaded to alginate and gum acacia needs further investigation. This study aimed to evaluate pharmacokinetic parameters using rabbits via oral administration.

**Experimental approach:**

The drug was administered orally to 2 groups of rabbits: pure ketoconazole (KTZ) and formulation of ketoconazole (AG75) groups. Blood samples were obtained from the ear marginal vein at various time points: 0 (before administration), 15, 30, 45, 60, 90, 120, 150, 180, 240, 300, 360, and 420 minutes after oral dosage. The pharmacokinetic study employed a non-compartment analysis to calculate the area under the curve (AUC), the volume of distribution (*V*_d_ *F*^-1^), clearance (*Cl F*^-1^), maximum concentration (*C*_max_), and time to reach maximum concentration (*t*_max_). The data obtained from the parameter result was analyzed using the independent-sample T-test.

**Key result:**

The results of the KTZ group include AUC of 15.83±0.62 h μg mL^-1^, *V*_d_*F*^-1^ of 8.95±1.17 mL, *ClF*^-1^ of 3.45±0.3 mL h^-1^, *C*_max_ of 4.7±0.69 μg mL^-1^, and *t*_max_ of 1.67±0.17 h. The results of the AG75 group include AUC of 27.8±1.01 h μg mL^-1^, *V*_d_*F*^-1^ of 11.5±2.4 mL, *ClF*^-1^ of 2.15±0.11 mL h^-1^, *C*_max_ of 4.49±0.52 μg mL^-1^, and *t*_max_ of 2.5±0.5 h.

**Conclusion:**

The formulation incorporating ketoconazole beads resulted in a higher AUC_0-∞_ than the pure ketoconazole. This finding suggests that the created formulation has enhanced the bioavailability of ketoconazole.

## Introduction

Ketoconazole is categorized as the Biopharmaceutics Classification System (BCS) Class IIb due to its limited solubility. The substance's solubility increases in acidic conditions, such as low pH or the pH range of the stomach (pH 1 to 3) [1]. On the other hand, the solubility of ketoconazole will decrease under alkaline conditions, such as the intestinal pH level [2].

The phenomenon of supersaturation is triggered by the decrease in solubility resulting from the pH increase as the substances proceed from the stomach to the small intestine. Supersaturation is a state characterized by the concentration of the drug exceeding the solubility equilibrium. In the present circumstances, there is a considerable augmentation in the drug's solubility; nonetheless, it is imperative to understand that supersaturation exhibits inherent thermodynamic instability. Hence, when reaching its critical concentration, the drug undergoes nucleation, subsequently leading to precipitation. The occurrence of precipitation reduces concentration due to the expansion of particles originating from nuclei [3,4]. The presence of precipitation might influence the rate and amount of drug absorption within the gastrointestinal tract, thereby impacting the bioavailability of the therapy [5].

The combination of alginate:gum acacia with mass ratio 75:25 in previous studies showed good results in inhibiting ketoconazole precipitation, which can maintain the supersaturation phase for 60 minutes compared to pure ketoconazole, which is only 20 minutes. This interaction occurs through hydrogen bonding between the negative group of the polymer as a hydrogen group donor and the hydrogen receptor group on ketoconazole. Hydrogen bonding between drug molecules and polymers can increase the activation energy of nucleation, thus inhibiting nucleation and crystal growth [6]. Alginate and gum acacia can produce a hydrogel through cross-linking with Ca^2+^ using ionotropic gelation techniques [7]. The combination produces mechanical properties of the polymer network to form a stronger hydrogel. Gum acacia is a good material to improve the physico-chemical properties of alginate hydrogel beads. The addition of gum acacia to the alginate solution can reduce the side-by-side aggregation of the alginate egg-box structure due to the presence of Ca^2+^ [8]. The results of synergistic testing of the AG75 combination that has been carried out previously show a synergistic effect between alginate and gum acacia with a mass ratio of 75:25 [9].

However, the pharmacokinetic study of the ketoconazole beads loaded to alginate and gum acacia needs further investigation. Therefore, the aim of this research was to evaluate the pharmacokinetic parameter of ketoconazole-loaded alginat:gum acacia with a mass ratio of 75:25 produced and characterized in the previous study [10]. The characterization results show that ketoconazole was successfully loaded in the matrix polymer, the testing including differential scanning calorimetry (DSC), scanning electron microscopy (SEM), Fourier transform infrared (FT-IR), X-ray diffraction (XRD), swelling study, in vitro drug release study, and solubility determination.

## Experimental

### Materials

Ketoconazole and itraconazole standard were bought from BPOM, Indonesia. Ketoconazole's active component was obtained from Kimia Farma Corps, Indonesia. EDTA K3 2 mL (Golden Vac, China). The sterile water for injection was manufactured by Ikapharmindo, Indonesia. Deionized water was supplied from CV. Alfa Kimia. Acetonitrile gradient grades for HPLC (Merck, Germany), NaH_2_PO_2_ (Merck, Germany), and NaOH (Merck, Germany).

### Animals

Three male New Zealand white rabbits, aged between three to four months, with an average weight ranging from 2.5 to 3 kilograms, were bought from Terminal Kelinci, a reputable rabbit breeder in Yogyakarta, Indonesia. Three rabbits were housed in a controlled environment within the Laboratory animal building, specifically at the Pharmacology department of the Faculty of Pharmacy in Indonesia. The rabbits were subjected to a 12-hour light and dark cycle. Their diet consisted of Vital Rabbit chow, manufactured by Citrafeed in Indonesia, and they had unrestricted access to water.

### Blood sampling and sample preparation

The rabbits underwent an overnight fasting period before the commencement of the experiment. A dosage of 14 mg kg^-1^ of KTZ was supplied to the rabbits, similar to the human dosage of 400 mg of KTZ. The drugs were administered orally to the rabbit via a tube. Approximately 2 mL blood samples were obtained as aliquots from the ear marginal vein at various time points: 0 (before administration), 15, 30, 45, 60, 90, 120, 150, 180, 240, 300, 360, and 420 minutes after oral dosage. The sample was obtained and placed in a tube containing 3 mL of ethylenediaminetetraacetic acid (EDTA). The blood samples were centrifugated at a speed of 3000 rpm for 10 minutes to get plasma. The plasma sample was transferred to a 1.5 mL microtube as the supernatant. The specimens were stored at a temperature of -18 °C until they were subjected to analysis.

**Figure 1.**
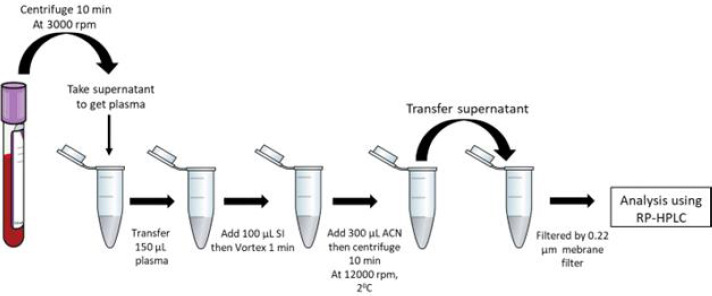
Preparation of blood plasma rabbit before analysis using RP-HPLC.

The addition of itraconazole as the internal standard (IS) involved the placement of 100 μL of the IS into a 1.5 mL microtube containing 150 μL of rabbit plasma sample. Subsequently, the sample was subjected to deproteinization using 300 μL of acetonitrile. Following vortexing for 1 minute, the plasma sample was centrifugated with 12000 g for 10 minutes. Subsequently, 100 μL of the resulting supernatant was carefully transferred to a sterile 1.5 mL microtube. Before conducting the analysis, the analyte underwent filtration using a membrane filter with a pore size of 0.45 μm. The concentration in the supernatant was assessed by employing the RP-HPLC technique for analysis (Figure 1).

### Analytical method

The method has been validated with the result of linearity in the 0.05-8 μg mL^-1^ with *R*^2^=0.9969 and LLOQ is 0.05 μg mL^-1^ [11]. The experimental setup included an Elite LaChrom high-performance liquid chromategraphy (HPLC) system, a Hitachi UV-Vis detector L-2420, and a Hitachi pump L-2130. A Phenomenex Luna column with dimensions of 250×4.6 mm and a particle size of 5 μm was utilized. The mobile phase utilized in this experiment was composed of a solution containing 0.02 M NaH_2_PO_4_ at a pH of 7.0, which was adjusted using 1 M NaOH. Additionally, acetonitrile was included in the mixture at a volume ratio of 30:70. The flow rate of the mobile phase was maintained at 1 mL min^-1^. The volume of the injection was 20 μL. The cellulose 0.45 μm membrane filter was employed to filter the mobile phase. The retention times for ketoconazole and itraconazole, used as standard internal compounds, were around 5 and 11 minutes, respectively.

### Data and statistical analysis

The pharmacokinetic study employed a non-compartment analysis using PK Analysis-2021R1 to calculate various pharmacokinetic parameters, including total area under the curve from time zero to infinity (AUC_0-∞_), the volume of distribution (*V*_d_ *F^-1^*), clearance (*Cl F^-1^*), maximum concentration (*C*_max_), and time to reach maximum concentration (*t*_max_).

The data obtained from the parameter resulted in an average ± standard error of measurement (SEM) and was analyzed using the Independent-Sample T-test. Statistically significant differences were seen when the p-value was less than 0.1.

## Results and discussion

**Figure 2.**
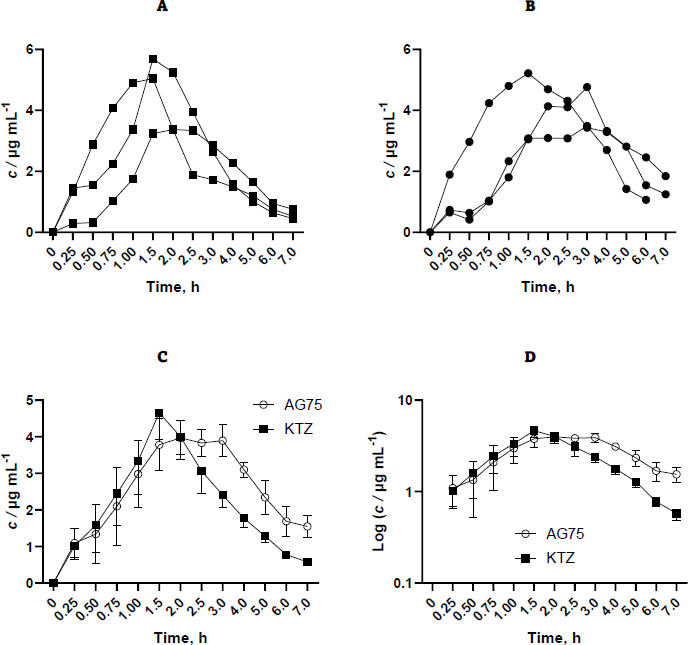
The concentration of ketoconazole in rabbit plasma by oral administration at a dose of 18.67 mg kg^-1^ in pure ketoconazole as control (A), beads as formula (B), overlapping of AG75 (alginate-gum acacia 75:25) and KTZ (ketoconazole) (C) and in semi-log graph (D). Mean data from *n*=3±SEM.

The materials employed in this investigation encompassed pure ketoconazole powder and AG75 ketoconazole beads. However, other ketoconazole beads were excluded from further pharmacokinetic evaluation due to their inability to impede ketoconazole precipitation. The administration of each sample was conducted by introducing it into the oral cavity of the rabbit by the utilization of a funnel/tube. Subsequently, a syringe supplemented 3-6 mL of water to the sample. The administered ketoconazole dosage was 400 mg, subsequently translated into a rabbit-specific dosage of 18.67 mg per kilogram of body weight. The selection of the 400 mg dose over the 200 mg dose was made in order to enhance the sensitivity of the testing device in detecting the drug concentration within the blood plasma matrix. Figure 2 shows the concentration results of purified ketoconazole and ketoconazole beads obtained from blood plasma at each sampling time.

Based on the available data, it is evident that variability exists across replicates, which can be attributed to physiological peculiarities inherent to each rabbit. Furthermore, administering drugs only through the oral route can lead to variations in the drug's entry time into the gastrointestinal tract, allowing for sufficient time for the rabbit to chew the beads before swallowing. Ideally, drug administration is performed with a catheter that enables direct oral administration into the gastric cavity. Nevertheless, the aperture proved insufficient in size, impeding the medicine from being discharged into the conduit. Inserting a bigger tube into the rabbit's stomach through the mouth cavity may provide challenges. Consequently, the drug was introduced via the oral route by use of the mouthpiece straight into the oral cavity of the rabbit. Table 1 displays the pharmacokinetic parameters examined, encompassing AUC_0-∞_, *C*_max_, *t*_max_, *V*_d_ *F*^-1^, and *Cl F*^-1^. The pharmacokinetic properties of the control drug and formula were examined and compared using an independent sample t-test at a confidence level of 90 %. The results demonstrated statistically significant differences in the AUC and *Cl F*^-1^ values. However, no statistically significant variances were seen in the remaining parameters.

**Table 1:** Pharmacokinetic parameters of ketoconazole in rabbit plasma by oral administration at a dose of 18.67 mg kg^-1^

Parameter	KTZ				AG75			
	#1	#2	#3	Average±SEM	#1	#2	#3	Average±SEM
AUC_0-∞_, h μg mL^-1^*	16.53	15.34	15.62	15.83±0.62	29.83	26.78	26.79	27.8±1.01
*C*_max_ /μg mL^-1^	5.69	5.04	3.37	4.7±0.69	4.76	5.22	3.49	4.49±0.52
*t*_max_ /h	1.5	1.5	2	1.67±0.17	3	1.5	3	2.5±0.5
*V*_d_ *F^-1^*/ mL	6.82	9.17	10.86	8.95±1.17	11.11	7.56	15.84	11.5±2.4
*Cl F^-1^*/ mL h^-1^ *	2.84	3.78	3.71	3.45±0.3	2.11	1.98	2.35	2.15±0.11

The parameters of pharmacokinetic are the area under the curve (AUC), volume of distribution (*V_d_ F^-1^*), clearance (*Cl F^-1^*), maximum concentration (*C*_max_), and time to reach maximum concentration (*t*_max_). The #1, #2, and #3 are means to replicate from the rabbits.

*significantly different (*p* < 0.1) between AG75 (alginate-gum acacia 75:25) and KTZ (ketoconazole). Mean data from *n* = 3±SEM.

Ketoconazole concentration data in blood plasma was processed using PK-Analysis software with non-compartment analysis. In this study, the formulation group produced higher *V*_d_ *F^-1^* values, from 8.95 to 11.5 mL, and lower *Cl F^-1^* values, from 3.45 hour to 2.15 mL h^-1^, compared to the control group.

The clearance of pure ketoconazole 400 mg in the form of a solution preparation has been studied previously by Huang *et al.* [12]. The results obtained a clearance of 2.05 mL h^-1^, which is not much different from the results in this study, which is 3.45 mL h^-1^. Clearance is defined as the clearance of a drug from plasma in a certain period. The process of reducing drugs from plasma can be through metabolism and elimination. Ketoconazole undergoes first-pass metabolism in the liver and gastrointestinal tract, which occurs extensively after absorption [12,13]. Ketoconazole is excreted in feces through bile as much as 20 to 65 %, while in urine, only 2 to 4 % [13,14]. The beads form of ketoconazole do not affect ketoconazole clearance.

The *V*_d_ *F^-1^* value is not significantly different between the control group and the formulation, so it can be said that the shape of ketoconazole beads does not affect the volume of distribution of ketoconazole. The volume of distribution depends on the binding in plasma and tissue, the accessibility of cells, and its partitioning into lipids [15]. The measured volume of distribution is the unbound form in plasma. Ketoconazole is 99 % bound and only 1 % free [16]. Because the drugs used are the same, the *V*_d_ *F^-1^* value of pure and formulated ketoconazole. Research on *V*_d_ *F*^-1^ ketoconazole with a dose of 400 mg has not been conducted before, so it cannot be compared.

The results of statistical testing of *Cl* values between control and formulation groups are significantly different. AUC is inversely proportional to clearance and is not significantly affected by the *V*_d_ *F*^-1^ value [17].

In the previous study, the *t*_max_ value of ketoconazole 400 mg tablets obtained were 1.7 h [18], 1.1 h [19], and 1.58 h [20], while for ketoconazole 400 mg solution dosage was 1.08 h [12]. In this study, the *t*_max_ obtained was 1.67 h for pure ketoconazole powder and 2.5 h for ketoconazole beads. The *t_max_* value of ketoconazole beads was greater than that of pure ketoconazole and tablets. It indicates that the sustained release of ketoconazole is released, so the time required to reach the maximum concentration is longer. However, the results of statistical testing of the control and formulation groups were not significantly different.

In this study, the *C*_max_ obtained was 4.7 μg mL^-1^ for pure ketoconazole powder and 4.49 μg mL^-1^ for ketoconazole beads. Both results were not significantly different (p>0.1). In the previous study, the *C*_max_ value of ketoconazole 400 mg tablets obtained was 7.08 μg mL^-1^ [18] and 5.71 μg mL^-1^ [20]. The *C*_max_ values of ketoconazole beads and pure ketoconazole have smaller values than previous studies. It is in line with the resulting AUC value and is also smaller. It might be because the drug administration to rabbits in this study was less than optimal. The drug that is inserted directly through the funnel has the potential not to be swallowed by rabbits. There is also the possibility of residual drug stuck to the tube wall to reduce the amount of drug administered.

## Conclusions

The pharmacokinetic profile of beads ketoconazole via oral route was evaluated. The formulation incorporating ketoconazole beads exhibited a higher AUC_0-∞_ than the pure ketoconazole. This finding suggests that the created formulation has enhanced the bioavailability of ketoconazole.
